# Multiple evidence for the role of an *Ovate*-like gene in determining fruit shape in pepper

**DOI:** 10.1186/1471-2229-11-46

**Published:** 2011-03-14

**Authors:** Aphrodite Tsaballa, Konstantinos Pasentsis, Nikos Darzentas, Athanasios S Tsaftaris

**Affiliations:** 1 Department of Genetics and Plant Breeding, School of Agriculture, Aristotle University of Thessaloniki, Thessaloniki, GR-541 24, Greece; 2 Institute of Agrobiotechnology (IN.A.), C.E.R.TH., 6th km Charilaou-Thermis Road, Thermi, GR-570 01, Greece

## Abstract

**Background:**

Grafting is a widely used technique contributing to sustainable and ecological production of many vegetables, but important fruit quality characters such as taste, aroma, texture and shape are known for years to be affected by grafting in important vegetables species including pepper. From all the characters affected, fruit shape is the most easily observed and measured. From research in tomato, fruit shape is known to be controlled by many QTLs but only few of them have larger effect on fruit shape variance. In this study we used pepper cultivars with different fruit shape to study the role of a pepper *Ovate*-like gene, *CaOvate*, which encodes a negative regulator protein that brings significant changes in tomato fruit shape.

**Results:**

We successfully cloned and characterized *Ovate*-like genes (designated as *CaOvate*) from two pepper cultivars of different fruit shape, cv. "Mytilini Round" and cv. "Piperaki Long", hereafter referred to as cv. "Round" and cv. "Long" after the shape of their mature fruits. The *CaOvate *consensus contains a 1008-bp ORF, encodes a 335 amino-acid polypeptide, shares 63% identity with the tomato OVATE protein and exhibits high similarity with OVATE sequences from other Solanaceae species, all placed in the same protein subfamily as outlined by expert sequence analysis. No significant structural differences were detected between the *CaOvate *genes obtained from the two cultivars. However, relative quantitative expression analysis showed that the expression of *CaOvate *followed a different developmental profile between the two cultivars, being higher in cv. "Round". Furthermore, down-regulation of *CaOvate *through VIGS in cv. "Round" changes its fruit to a more oblong form indicating that *CaOvate *is indeed involved in determining fruit shape in pepper, perhaps by negatively affecting the expression of its target gene, *CaGA20ox1*, also studied in this work.

**Conclusions:**

Herein, we clone, characterize and study *CaOvate *and *CaGA20ox1 *genes, very likely involved in shaping pepper fruit. The oblong phenotype of the fruits in a plant of cv. "Round", where we observed a significant reduction in the expression levels of *CaOvate*, resembled the change in shape that takes place by grafting the round-fruited cultivar cv. "Round" onto the long-fruited pepper cultivar cv. "Long". Understanding the role of *CaOvate *and *CaGA20ox1*, as well as of other genes like *Sun *also involved in controlling fruit shape in Solanaceae plants like tomato, pave the way to better understand the molecular mechanisms involved in controlling fruit shape in Solanaceae plants in general, and pepper in particular, as well as the changes in fruit quality induced after grafting and perhaps the ways to mitigate them.

## Background

Fruit shape is an easy to observe and measure, quantitatively inherited character. In tomato (*S. lycopersicum*) fruit shape is controlled by many Quantitative Trait Loci (QTLs) but only few of them attribute mostly to variance: *Ovate*, *Sun*, *Fruit Shape (Fs) 8.1 *and *Triangle (Tri) 2.1*/*Blockiness *(*Dblk) 2.1 *[1]. The first of these loci, *Ovate*, is a major QTL that as was shown first in tomato, encodes a negative regulator of fruit elongation protein, acting early in flower and fruit development [2]. A single mutation creating a stop codon in the second exon of the coding sequence of *Ovate *differentiates the pear-shaped or elongated from the round-shaped tomato fruit [2]. The mutation in *Ovate *sequence is not linked to a single phenotype: depending on the genetic background, the extent of fruit elongation, as a result of the fruit's neck constriction, is more or less distinct [3].

Recent studies in *Arabidopsis *implicated a second member of the OVATE Family of Proteins (OFPs), AtOFP1, to the regulation of cell elongation, by actually suppressing *AtGA20ox1*, a gene that encodes a critical enzyme in the gibberilin (GA) pathway [4]. AtOFP1 exerts its function through binding to KNAT1 [5], a member of the KNOTTED1-like homeodomain (KNOX) family of proteins already known repressors of *GA20ox1 *transcription [6,7]. *GA20ox1 *that catalyzes the conversion from GA_19 _to GA_20_, determines the production of GA, a plant hormone that promotes a large number of physiological processes such as stem, root, stamen, pistil, leaf and hypocotyl elongation in a variety of plants [8]. Lately, in *Arabidopsis*, it was shown that AtOFP1 interacts with AtKU, a protein with multiple functions, being involved in the DNA repair also through the non-homologous end-joining pathway [9], consistent with previous suggestions that AtOFP1 may control the expression of other genes, besides *AtGA20ox1*[5]. AtOFP1 and AtOFP5, were shown to be located in the cytoskeleton and direct the movement of a member of BELL proteins family, BLH1 (another homeodomain containing transcription factor), from the nucleus to the cytoplasm, thus preventing its action as transcription factor [10]. KNOX and BELL homeodomain proteins belong to the TALE (**T**hree-**A**mino-acid **L**oop **E**xtension) protein superfamily and they interact [10-14] forming heterodimers. The action of such a BELL-KNOX heterodimer was shown to be negatively regulated by AtOFP5 ensuring normal embryo sac growth in *Arabidopsis *[15]. On the other hand, potato TALE proteins, StBEL5 and POTH1, were shown to interact and bind to a specific 10-bp sequence of the promoter of *GA20ox1 *[16].

In pepper (*C. annuum*) it was also shown that fruit shape is controlled by few major QTLs [17,18]. To gain insight on the molecular mechanisms involved in the determination of fruit shape in pepper, we have cloned and characterized the full length cDNA of *CaOvate *from a round fruit shaped pepper cultivar (cv.), named cv. "Round", by reverse transcriptase polymerase chain reaction (RT-PCR). We then cloned the corresponding genomic fragments from cv. "Round" and another pepper cultivar, with long shaped fruits, named cv. "Long" and studied *CaOvate *in both cultivars. Real time PCR was used for relative quantitative comparative expression analysis in various stages of flower and fruit development in these two cultivars. Critically, we successfully silenced *CaOvate *in cv. "Round" plants using the Tobacco Rattle Virus (TRV) -based Virus-Induced Gene-Silencing (VIGS) system which resulted in obvious change of fruit shape, followed by an increase in the expression of *CaOvate*'s target gene, *CaGA20ox1*. We finally present our conclusions and discuss implications and future directions.

To the best of our knowledge, this is the first report of genes involved in shaping pepper fruit, a character known for years to be affected by grafting [19-21]. In conjunction with the remarkable progress in genomic sequencing of many Solanaceae species such as pepper and other complementary -omic studies, we believe our work is a step forward in better understanding the molecular mechanisms involved in controlling fruit shape in pepper.

## Methods

### Plant material

Seeds from two *C. annuum *cultivars, cv. "Mytilini Round" (referred to from now on as cv. "Round") and cv. "Piperaki Long" (referred to from now on as cv."Long") were used in this study. The fruits of cv. "Round" are spherical in shape and pendent, while the fruits of cv. "Long" are oblong and erect. The seeds from both cultivars were initially sown in small pots up to stage of 3 to 4 true leaves. All seedlings were transplanted in bigger pots, in 3:1 mixture of soil and perlite. Frequent fertilization was supplied as 20 units total N_2_, 20 units P_2_O_5 _and 20 units K_2_O. The plants were grown in a growth chamber under a photoperiod of 16 hr light (25°C) and 8 hr dark (20°C).

### RNA isolation and cDNA synthesis

Samples from buds before anthesis (4-5 DBA), open flowers, ovaries of open flowers, 5 days after anthesis (5 DAA) and 10 days after anthesis (10 DAA) developing fruit, and early fruit were collected from several plants of cv. "Round" and cv. "Long", immediately frozen in liquid nitrogen and stored at -80°C for a maximum of 4-5 days. Total RNA was extracted using the TRIzol reagent according to the manufacturer's instructions (Invitrogen, Carlsbad, CA, USA). The quantity and purity of the extracted total RNA was measured by spectrophotometry while the quality and integrity was estimated by gel electrophoresis.

First strand cDNA was synthesized from 1 μg of each total RNA, using 0.5 mM dNTPs, 1× First-Strand Buffer, 10 mM DTT, 200 Units (U) SuperScript II Reverse Transcriptase (Invitrogen) and 250 ng random hexamers or 0.5 μgr of the 3' RACE Adapter Primer (5'-GGCCACGCGTCGACTAGTAC(T)_17_-3') (Invitrogen), in 20 μl total volume, according to the manufacturer's protocol.

### Cloning of *Ovate *gene from pepper

The tomato *Ovate *gene [GenBank: AAN17752.1], was used in a BLAST search at NCBI [22], to identify similar sequences from pepper, and a *C. frutescens *BAC genomic clone [BAC 215H17, GenBank: EF517792] with high similarity was obtained. In order to verify mRNA expression of this putative gene and the length of 3' Untranslated Region (UTR), primer OVATE FOR 1 (for all primers' sequences see Additional File 1) was specifically designed according to the sequence of the BAC clone (position from 32356 to 32374) and used in the subsequent 3' RACE experiments. 1 μl of the cDNA from cv. "Round" open flowers, synthesized with the 3' RACE Adapter Primer (as described above), was used as a template in a PCR reaction with 0.5 μM primers OVATE FOR 1 and Abridged Universal Amplification Primer (AUAP), 0.2 mM dNTPs and 1 U of DyNAzymeII DNA polymerase (Finnzymes, Espoo, Finland) in 50 μl reaction volume. The thermocycler program was 2 min at 94°C; 30 cycles of 30 s at 94°C, 30 s at 52°C, 30 s at 72°C and a final extension step of 10 min at 72°C. A product of about 250-bp was purified from the gel using the Nucleospin - Extract II kit (Macherey - Nagel, Germany) and cloned into the pCR II-TOPO vector (Invitrogen) according to the manufacturer's protocol. Five individual clones were commercially sequenced. Sequencing results were analyzed using the DNASTAR software (DNASTAR, Madison, WI). It was confirmed that all clones contained the appropriate fragment.

Based on this information, a pair of new primers, OVATE FOR 2 and OVATE FINAL, was designed and used to amplify the whole coding sequence of *Ovate *from *C. annuum *pepper cv. "Round". 1 μl of the synthesized, with random hexamers, cDNA from cv. "Round" open flowers, served as template in a PCR reaction, in which 0.5 μΜ of gene-specific primers, 0.2 mM dNTPs and 1 U DyNAzyme II DNA polymerase (Finnzymes) were used. The thermocycler program was 35 cycles of: 30 s at 94°C, 30 s at 52°, and 1 min at 72°C, which were preceded by 5 min at 94°C and followed by 10 min at 72°C. Amplified fragments were cloned into a pCR II-TOPO vector (Invitrogen) and commercially sequenced. Sequencing results, analyzed as above, revealed that the clones contained the full-length coding sequence of *Ovate*, designated from now on as *CaOvate *[GenBank: JF427571].

### DNA isolation, amplification of *CaOvate* gene and isolation of 5' upstream sequences

Total genomic DNA was isolated from leaves of cv. "Round" and cv. "Long" using the standard C.T.A.B protocol [23]. DNA quantity was measured by spectrophotometry.

For the amplification of the whole *CaOvate *gene from cv. "Round" and cv. "Long", 50 ng of genomic DNA were used as a template in a PCR reaction using 0.5 μΜ of primers OVATE FOR 2 and OVATE FINAL, 0.2 mM dNTPs and 1 U DyNAzyme II DNA polymerase (Finnzymes). The thermocycler program was 35 cycles of: 30 s at 94°C, 30 s at 52° and 1 min at 72°C, which were preceded by 5 min at 94°C and followed by 10 min at 72°C. Amplified fragments were cloned and the resulting clones were sequenced and analyzed as above. The genomic sequences *CaOvate *obtained from both cultivars along with the genomic sequence of the *C. frutescens *BAC clone, were aligned using the ClustalW2 multiple sequence alignment program [24]. The alignment was edited with Bioedit [25].

For the isolation of 5' upstream sequences of *CaOvate*, the Rolling Circle Amplification of Genomic templates for Inverse PCR technique (RCA-GIP) was employed as described by [26]. Briefly, one μg of genomic DNA from cv. "Long" was digested, in independent reactions, with three restriction enzymes, *EcoRI*, *XbaI *and *XhoI *(New England Biolabs, Ipswich, MA, USA) in a total volume of 25 μl. Self-ligation and *φ*29 DNA polymerase (New England Biolabs) amplification of this circular genomic DNA followed. Inverse PCR reactions were performed using as template 1 μl of an 1:100 dilution of the rolling circle amplification reactions, 0.2 μM of gene specific primers for *CaOvate*, OVATE FOR 3 and OVATE REV 1 and 1 U DyNAzyme II DNA Polymerase (Finnzymes). The thermocycler conditions were 2 min at 94°C; 30 cycles of 20 s at 94°C, 30 s at 58°C, 2 min at 72°C and a final extension step of 10 min at 72°C. The RCA template from the *XbaI *digest library produced an amplified product of about 3500-bp that was directly purified using the Nucleospin - Extract II kit (Macherey - Nagel). Cloning into the pCR II-TOPO vector (Invitrogen) and sequencing followed until finally one contig was assembled. Based on these sequencing results a primer (OVATE FOR 5) was designed and used along with primer OVATE REV1, for the amplification of a fragment belonging to the 5' upstream region from cv. "Round", which was sequenced too.

### Protein sequence comparisons and phylogenetic analysis of CaOVATE

The deduced amino-acid sequence of *CaOvate *was used for a search in the Pfam 24.0 database [27] and the Pfam domain DUF623 [Pfam: PF 04844] was detected. Following the identification of this conserved domain, we collected all *Viridiplantae *proteins from Pfam and UniProt [28] databases with a statistically significant hit for the DUF623 domain. All the sequences collected were aligned using MAFFT, a multiple sequence alignment program [29]. The resulting alignment was edited with Jalview [30] and subjected to extensive manual curation removing columns having many gap characters. This curated alignment was used for protein subfamily identification employing the SCI-PHY algorithm [31]. After subfamily identification, the multi-RELIEF Feature Weighting Method [32] was employed to detect specificity determining amino-acid residues among subfamilies. For the phylogenetic analysis the MAFFT program was also used. The resulting tree was edited with the Figtree v1.3.1 software [33].

In an attempt to retrieve sequences homologous to *CaOvate *from more Solanaceae species and therefore study the phylogenetic depth of our sequence, we performed extensive BLAST searches using recent (Release 106 December 2010) and comprehensive plant-specific nucleotide sequence data from EMBL-EBI [34] with our sequence as query and an e-value of 1e-20. The databases used were the EST (Expressed Sequence Tags), GSS (Genome Survey sequences), HTC (High throughput cDNA sequencing), HTG (High Throughput Genome sequencing), CDS (Coding sequences) and STD (Standard - all entries not classified as above).

### Expression analysis of *CaOvate*

Relative quantitative expression analysis of *CaOvate *during flower and fruit development for the two cultivars, cv. "Round" and cv. "Long", was performed with real-time RT-PCR using a Rotor Gene 6000 (Corbett, Australia) real-time PCR system. OVATE FOR 3 and REV 2 was the primer pair used, with the forward primer specifically used due to its design in the first exon - intron junction to avoid amplification of genomic DNA. The PCR was performed in 1× Platinum SYBR Green qPCR SuperMix - UDG (Invitrogen) containing 0.5 μM of each primer and the template was 1/10 of the cDNA, synthesized with random hexamers, from RNA extracted from: (a) buds (4-5 DBA), (b) ovaries of open flowers, (c) 5 DAA and 10 DAA developing fruits and (d) early fruits. The cycling parameters were: incubation at 50°C for 2 min, 95°C for 2 min, followed by 35 cycles of incubation at 95°C for 20 s, 58°C for 20 s, 72°C for 20 s, and a final extension step of 10 min at 72°C. To identify the PCR products, a melting curve was performed from 65 to 95°C with observation every 0.2°C and a 5 s hold between observations. The reactions were performed in triplicate. Relative quantification and statistical analysis were performed using the LinRegPCR software version 11.1 [35], which is using the linear regression analysis to calculate the starting concentrations of mRNA's and individual PCR efficiencies for each sample. *CaOvate *expression was normalized against the non regulated reference gene pepper *Actin *[GenBank: AY572427]. Primers for pepper *Actin *were adapted from [36].

### Virus Induced gene Silencing of *CaOvate*

#### Plasmid construction

pTRV1, pTRV2 vectors and pTRV2-*Nicotiana benthamiana *(Nb) *Phytoene Desaturare *(*Pds*) construct were provided by the Arabidopsis Biological Resource Center (ABRC) [37] and have been described previously [2].

For the constructs' assembly, a pCR II-TOPO cDNA *CaOvate *clone, already verified by sequencing that contains a 962-bp fragment of the mRNA of the gene (from position 1 to position 962 of the mRNA of the *CaOvate*), was *EcoRI *digested. The digestion produced a 794-bp fragment that lacked 168-bp of the 5' of the mRNA (from position 1 to position 168), due to an additional, inside the initial 962-bp fragment, *EcoRI *site. This 794-bp fragment was then ligated to the pTRV2 vector, already digested with *EcoRI *and dephosphorylated, using 1 U of T4 DNA ligase (Invitrogen) in 1× Ligase Reaction Buffer. 1 μl of the ligation reaction was used for the transformation of Mach1-T1 competent cells (Invitrogen) via electroporation (MicroPulser electroporator, Bio-Rad Laboratories, Inc.). All constructs were verified by restriction digestion and sequencing.

#### Agro-infiltration

Initially, in order to test the effectiveness and the efficiency of VIGS in cv. "Round" plants, a test experiment for silencing of the *Pds *gene was carried out. Plants of cv. "Round" were grown in pots at 24°C in a growth chamber under 16 hr light/8 hr dark cycle with 60-70% humidity. For the agro-infiltration, pTRV1, pTRV2 (empty vector), and pTRV2-*NbPds*, were transformed into *Agrobacterium tumefaciens *GV3101 via electroporation. Each strain was grown in 5 ml LB (supplemented with 50 mg/ml of kanamycin and 50 mg/ml of gentamycin) overnight at 30°C. The overnight culture was inoculated into 50 ml of LB medium and grown at 30°C overnight. *Agrobacterium *cells were harvested by centrifugation (2000 g, 20 min, 15°C), resuspended in infiltration medium (10 mM MES, 200 μM acetosyringone, 10 mM MgCl_2_), and adjusted to an O.D_600 _of 1.6-1.8. The cultures were then left at room temperature for 3-4 hr. *Agrobacterium *cells carrying pTRV1 and pTRV2 or pTRV2-*NbPds *(1:1 ratio) were infiltrated by pressuring a needle-less syringe into the cotyledons of pepper seedlings. The plants were covered and left like this overnight. Three weeks later the majority of the plants infiltrated, exhibited extensive photobleaching in their leaves. It was observed that infiltrated plants kept on producing photobleached white leaves even four months after the infiltration. Plants infiltrated with pTRV1 and pTRV2 (empty vector) didn't exhibit photobleaching.

For the VIGS of *CaOvate *the procedure followed was the same as described above. After the infiltrations, plants of cv. "Round" agro-infiltrated with pTRV1, pTRV2 (empty vector) and the recombinant plasmids pTRV2-*CaOvate *sense and pTRV2-*CaOvate *antisense (1:1 ratio) were transplanted after a while into bigger pots and frequently fertilized thereafter.

#### RT-PCR analysis of *CaOvate*

To investigate the expression of endogenous mRNA *CaOvate *in *CaOvate*-silenced plants, total RNA was extracted from leaves and small fruits, and first-strand cDNA synthesis was carried out, as described above, using random hexamers. For the viral RNA detection, through RT-PCR, specific primers were used. For TRV1 detection, primer TRV1 FOR was designed specifically on the TRV segment RNA1 complete sequence [GenBank: AF406990] (from position 5979 to 5998) while primer OYL 198 REV was adapted from [38]. Primers for TRV2 detection were designed on the coat protein region of TRV RNA2-based VIGS vector pTRV2 [GenBank: AF406991] (Coat Protein FOR: position 800 to 819, Coat Protein REV: position 915 to 933). To distinguish between amplification of the endogenous mRNA transcripts of *CaOvate *from the viral-derived ones, one of the two primers used in the RT-PCR experiments came from the 3' UTR area of the gene outside the region used in the pTRV2 constructs (primer OVATE FINAL). The other one (primer OVATE FOR 4) was designed in position 621 to 641 of the mRNA of *CaOvate*. The real time RT-PCR was performed as described in the *Expression analysis of CaOvate *section with the only exception the different cycling parameters which were: incubation at 50°C for 2 min, 95°C for 2 min, followed by 35 cycles of incubation at 95°C for 20 s, 58°C for 20 s, 72°C for 20 s, and a final extension step of 10 min at 72°C.

In order to identify possible effects of *CaOvate *silencing in the expression of its target gene, *GA20ox1*, we acquired a putative *GA20ox1 *gene from pepper. Using the tomato *GA20ox1 *sequence [GenBank: EU043161], in a BLAST search, one EST [GenBank: GD070135] was retrieved from the Pepper EST database [39]. Employing the RCA-GIP technique [26] we were able to acquire the full length genomic *GA20ox1 *sequence from cv. "Long" (designated as *CaGA2ox1*) [GenBank: JF427572], including the missing, from the initial EST, 5' end. For the relative quantification of *CaGA20ox1 *expression levels of the infiltrated plants by real time RT-PCR, primers GA20ox1 FOR 2 and REV 2 were designed, based on the sequence information obtained from RCA-GIP experiments and the presumable intron-exon organization of the gene. The cycling parameters were: 50°C for 2 min, 95°C for 2 min, followed by 35 cycles of incubation at 95°C for 20 s, 58°C for 20 s, 72°C for 25 s, and a final extension step of 10 min at 72°C.

## Results

### Cloning of *CaOvate*

A 3' RACE approach was used along with an *Ovate *gene-specific primer, OVATE FOR 1 (for all primers' sequences see Additional File 1), designed on a specific region identified by BLAST, of a *C. frutescens *BAC clone genomic sequence to obtain a full-length *CaOvate *cDNA. The resulting cDNA fragment was isolated, cloned and sequenced. All clones were identified as *CaOvate *using BLAST. Based on this information a new primer pair was designed (OVATE FOR 2 and OVATE FINAL) which was used in a PCR to produce full-length cDNA *CaOvate *clones from cv. "Round". From the individual clones analyzed using the SeqMan software package (DNA Star, Madison, WI), a single contig of 1116-bp was produced, that contained a 1008-bp ORF encoding a 335 amino-acid polypeptide. The alignment of the *CaOvate *cDNA sequence from cv. "Round" to the one from the genomic BAC clone of *C. frutescens *showed that there was only one nucleotide difference between the two sequences, in position 419 of the cDNA.

**Figure 1:**
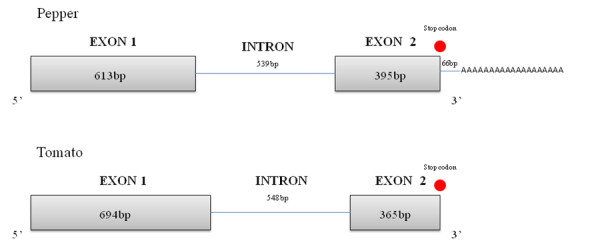
**Genomic organization of *Ovate *genes from pepper (*CaOvate*) and tomato**. As is shown on the top, the gene in pepper has two exons of 613- and 395-bp and a single intron of 539-bp. In addition the stop codon is indicated just before the 3' UTR of 66-bp, followed by the poly -A tail. At the bottom, the corresponding gene in tomato has a similar organization two exons of 694- and 365-bp and an intron of 548-bp.

The aforementioned alignment also provided hints about the genomic organization of the *CaOvate *gene. In order to verify this, OVATE FINAL was used, along with the primer OVATE FOR 2 to obtain the genomic sequence of *CaOvate *gene from DNA extracted from young leaves of cv. "Round". A PCR fragment of 1570-bp was purified from the gel, cloned in a pCR-II TOPO vector and sequenced. One contig was assembled that contains the whole coding genomic sequence of *CaOvate *from cv. "Round". Using this coding genomic sequence and the Splign program at NCBI, we observed that the genomic organization of *CaOvate *consists, as it was predicted, of two exons, the first and larger of 613-bp and the second, and smaller, of 395-bp. The unique intron of the gene consists of 539-bp. After the stop codon, a 3' UTR of 66-bp and poly-A tail follow. The genomic organization is conserved in the *Ovate *gene from tomato, where two exons of 694-bp and 365-bp, respectively, are interrupted by an intron of 548-bp (Figure 1).

To examine whether genetic changes within the *CaOvate *sequence are responsible for the differences in the shape of the two pepper cultivars, we obtained the genomic sequence of *CaOvate *from cv. "Long", with the elongated fruit shape. The analysis of the genomic sequence of *CaOvate *from cv. "Long" revealed one Single Nucleotide Polymorphism (SNP) located in the translated region of the first exon, position 419 resulting in a cytosine in cv. "Round" to guanine substitution in cv. "Long". This replacement changes the ORF of the sequence resulting in a Threonine_Long _- to - Serine_Round _polymorphism. However this change is not considered to be decisive since threonine and serine are biochemically similar amino-acids. Another SNP is located inside the intron, in position 746. Both sequences from the cultivars were aligned to the genomic sequence of the *C. frutescens *BAC clone. *CaOvate *sequence from cv. "Long" is almost identical to the one from *C. frutescens*, with the exception of one nucleotide change but in the intron area (position 654). *CaOvate *sequence from cv. "Round" differs from the sequence of *C. frutescens *in the same positions as with cv. "Long" (positions 419 and 746) plus position 654 (see Additional File 2).

### Amino-acid sequence and phylogenetic analysis of CaOVATE

**Figure 2:**
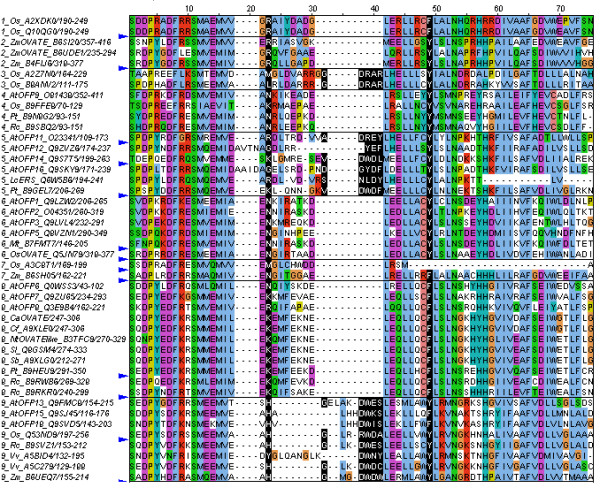
**Multiple alignment of DUF 623 domains from a number of OFPs**. Sequences come from the family of Solanaceae (*S. lycopersicum - Sl, N. tabaccum- Nt*, *S. bulbocastanum - Sb, C. annuum - Ca, C. frutescens - Cf*), *A. thaliana *(AtOFPs), *Z. mays *(Zm) and *O. sativa *(Os) as well as from putative orthologs from the complete plant section of the Uniprot database. The alignment was generated using the MAFFT program and edited with Jalview. The name of each sequence consists of the number of subfamily, followed by the species, its characterization in the databases (if exists) and the Uniprot ID. Identically colored amino-acids share similar biochemical properties. Informative residues identified with the multi-RELIEF algorithm are highlighted in black background. Several protein sequences (indicated by small blue wedges) have been hidden for clarity.

We collected sequences of proteins homologous to the CaOVATE predicted protein sequence as described in *Methods*. All of the proteins retrieved share a C terminal domain, DUF623 [Pfam: PF04844], which is an uncharacterized domain of about 70 residues found exclusively in plants. The multiple alignment of all the sequences highlights interesting features including the near perfect conservation of the DUF623 domain inside the Solanaceae family (Figure 2). The conservation across the alignment is higher in the beginning (position 1 to 17) and in the end of the domain (position 42 to 69). Amino-acids that appear to be very highly conserved (> 95%) across sequences are: the proline at position 4, the phenylalanine at position 8, the serine at position 11, the methionine at position 15, the leucine at position 46, the asparagine at position 53, the isoleucine at position 61 and finally the phenylalanine at position 65.

Using the SCI-PHY algorithm (see *Methods*), nine subfamilies (subf.) were identified. All the Solanaceae OVATEs are included in one subfamily (subf. 8) along with *Arabidopsis thaliana *(At) OFP6 [Uniprot: Q0WSS3], AtOFP7 [Q9ZU65], AtOFP8 [Q3E9B4]. AtOFP1 [Q9LZW2], AtOFP2 [O04351], AtOFP3 [Q9LVL4] and AtOFP5 [Q8VZN1] are categorized in another subfamily (subf. 6) along with the OVATE-like from *O. sativa *[Q5JN79]. OVATEs from *Z. mays *[B6UDE1 and B6SI20] are placed in subf. 2. The other *Arabidopsis *OFPs are grouped into two more subfamilies: subf. 5 which includes AtOFP11 [O23341], AtOFP12 [Q9ZVZ6], AtOFP14 [Q9S775], AtOFP16 [Q9SKV9] and subf. 9 which includes AtOFP13 [Q9FMC8], AtOFP15 [Q9SJ45], AtOFP18 [Q9SVD5]. In subf. 5 the domain of Ethylene Receptor (ERS) from *L. chinensis *[Q6W5B6] is included. In all subfamilies DUF623 domains of predicted or putative proteins from other species such as *V. vinifera*, *P. trichocarpa*, R. com*munis*, *O. sativa*, *Z. mays *etc are included (Figure 2). There are many potential specificity determining residues, i.e. capable of separating the subfamilies, that can be seen highlighted in black background at alignment positions 23, 32, 38, 39, 40, 41 and 49. More specifically, in position 49, the polar amino-acid tyrosine in subf. 5, 2, 6 and 9 (apart from sequences AtOFP15 and AtOFP18) is substituted by a hydrophobic, non polar, phenylalanine in subf. 8 and subf. 1. Positions 32, 38, 39, 40 and 41 of the alignment are occupied by amino-acids only in subf. 9, 5 and 3. Finally, in subf. 8, position 23 is either lysine (Solanaceae OVATEs) or arginine, which are biochemically similar amino-acids (the only exception being AtOFP6 which contains asparagine). In subf. 6 the corresponding amino-acid in position 23 is mainly asparagine while in subf. 2 is arginine. The amino-acid in this position in subf. 9 is mainly histidine and in subf. 5 is either glycine, lysine, or arginine (the last two being biochemically similar).

A phylogenetic tree was also calculated based on the alignment generated by the MAFFT program. The tree depicts the phylogenetic distance between the subfamilies, determined by SCI-PHY. Close to subf. 8 in which the OVATEs from the Solanaceae are included, are subf. 7, subf. 2, in which the *Z. mays *OVATEs are incorporated, subf. 4 and subf. 6 with all the previous characterized AtOFPs such as AtOFP1 and AtOFP5 (see Additional File 3).

The *CaOvate *cDNA sequence was then used in extensive BLAST searches against recent and comprehensive plant nucleotide sequence databases in order to identify further homologies especially among species of the Solanaceae family. Indeed, several hits were ESTs of new - compared to the alignment of Figure 2 - Solanaceous plants like eggplant (*S. melongena*) and chaco potato (*S. chacoense*), while we also recovered a genomic sequence from *S. phureja*, another new addition to the list of species our sequence apparently has homologs in. On top of this, and as expected, numerous hits in different databases were found of plants already present in our primary bioinformatics analysis. Overall, these results (Additional File 4) provide supporting and additional evidence that the *CaOvate *sequence is deeply conserved in the Solanaceae family, thus possibly functionally relevant and potentially useful for further research and biotechnological applications.

### Expression analysis of *CaOvate*

**Figure 3:**
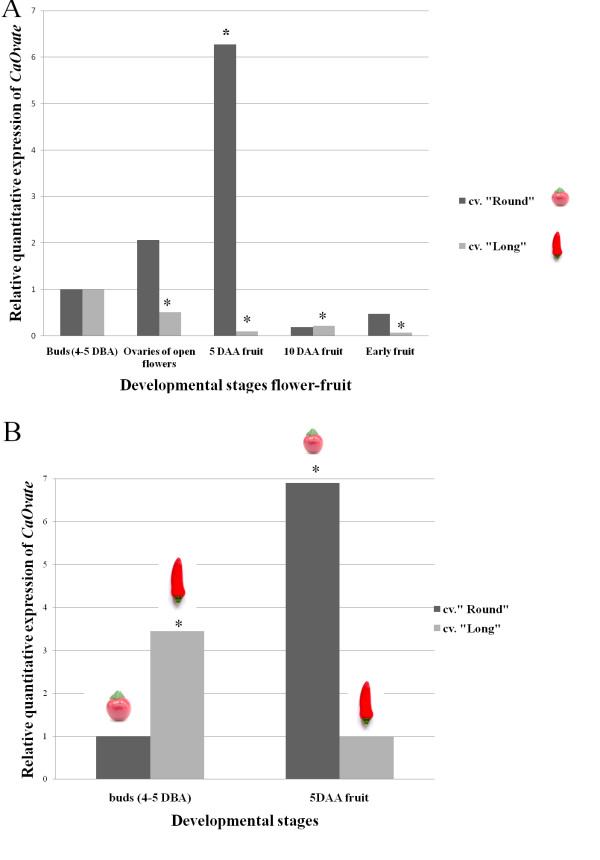
**Expression analysis of *CaOvate *in different stages of flower and fruit development of cv. "Round" and cv. "Long"**. A) Relative quantitative analysis of *CaOvate *expression. Sampling was from 4-5 DBA (buds) until the end of fruit development (early fruit). The relative expression ratio in each sample in comparison with the control sample, which was in both cultivars buds of 4-5 DBA, is represented by a factor of up- or down- regulation and is shown with bars for the cultivar "Round" and "Long". During flower's and fruit's development, *CaOvate *expression follows different developmental expression patterns in the two cultivars: in cv. "Round" the expression reaches is highest after anthesis while in cv. "Long" the highest expression is demonstrated before anthesis (data derive from two independent real-time RT-PCR experiments). B) New relative quantitative analysis of *CaOvate *expression in two specific developmental stages: before anthesis (4-5 DBA) where the gene exhibits its higher expression in cv. "Long", and after anthesis (5 DAA), where the gene exhibits its higher expression in cv. "Round". The relative expression ratio, represented by a factor of up- or down- regulation, is shown with bars for the cultivar in each sample and in comparison with the control sample, which in buds was the one from cv. "Round" while in 5 DAA fruit was the one from cv. "Long". Asterisks indicate statistically significant difference (p < .05) of the each sample compared to the corresponding control sample.

The *Ovate *in tomato is expressed in the reproductive organs in early stages of flower and fruit development [2]. *Ovate *transcripts can be detected in flowers 10 days before anthesis (DBA) and until 8 days after anthesis (DAA) in developing fruit, at which time *Ovate *transcript levels begin to decrease [2]. To test whether this developmental expression profile is the same in pepper, real time PCR experiments were performed to determine expression levels of the *CaOvate*, on cDNAs derived from tissues of several flower and fruit developmental stages of cv. "Round" and cv. "Long". The highest expression of *CaOvate *in cv. "Round" is exhibited after anthesis, and specifically in the 5 DAA developing fruit. Before this peak the expression of *CaOvate *is lower while after the peak the transcript level drops to a nearly undetectable level (Figure 3A). On the contrary, *CaOvate *expression in cv. "Long" follows a different developmental profile: the highest expression is exhibited before anthesis, in the buds of 4-5 DBA and falls sharply afterwards. Thus at the stages of buds at 4-5 DBA and 5 DAA, where cv. "Long" and cv. "Round" exhibit a peak of *CaOvate *expression respectively, large differences are observed. To quantify these differences more accurately, a new real time PCR experiment was conducted, including the two stages of buds 4-5 DBA and developing fruit 5 DAA. In buds the expression of *CaOvate *in cv. "Long" is higher than in cv. "Round". However in the developing fruit of 5DAA the expression of *CaOvate *in cv. "Round" is higher than in cv. "Long" and actually even higher than in every other sample-developmental stage tested (Figure 3B).

### Isolation of 5' upstream sequences

In order to explore if genetic changes in the 5' upstream region of *CaOvate *in the two cultivars are responsible for any differences in the expression levels of *CaOvate*, we acquired a considerably large fragment of this region (~2500-bp) from applying the RCA-GIP technique [26] in cv. "Long". Next the corresponding region was amplified from cv. "Round". The sequences obtained by the two cultivars included only minimum differences; only a SNP was spotted in pos. -1526 upstream of the start codon. The comparison of both cultivars sequences to the sequence of the *C. frutescens *BAC clone, demonstrated 5 SNPs in a region approx. -1000 from the start codon, corresponding to the probable promoter region of the gene. The role, if any, of these SNPs in binding sites of regulatory elements remains to be studied.

### VIGS of *CaOvate *in cv. "Round"

In order to obtain further evidence for the role of *CaOvate *in determining fruit shape in cv. "Round", the VIGS technique was used. VIGS of the *Pds *gene was used as a control resulting in photobleaching that was obvious in the majority of pepper plants infected and persisted even 4 months after the infiltration. Photobleached leaves were collected and used as control in the experiments described below. For VIGS constructs with *CaOvate*, a 794-bp fragment was used, that contained the part of the cDNA sequence also coding for the DUF623 domain. The choice of including this part of the gene was consistent with the idea to simulate by VIGS what seems to be the case in tomato, where the disruption of the second exon by a stop codon causes the abolishment of the DUF623 domain and thus the change in fruit shape [2].

Firstly, in a preliminary experiment to determine the appropriate developmental stage for applying the VIGS technique, a small number of cv. "Round" pepper plants was infiltrated at the stage of 4-5 true leaves, with *Agrobacterium *cells harboring pTRV2-*CaOvate *sense or pTRV2-*CaOvate *antisense and one plant with pTRV1 and pTRV2 (empty vector). Approximately 2 months after the infiltration and while the plants were developing numerous fruits, it was noticed that in a specific plant (infiltrated with pTRV2-*CaOvate *sense), fruits that exhibited a more oblong shape were co-developing next to fruits that exhibited the typical round shape of the cultivar cv. "Round". The phenotypic measurements of the mature fruits of this plant showed a statistically significant change in fruits' length and consequently in fruit shape index (the ratio of highest fruit height to widest width) compared to that of the wild type (Additional File 5). This spatial expression of the VIGS phenotype is a phenomenon also noticed before by Rotenberg et al [40], working with tomato. Furthermore, following the findings of Chung et al [41] that for chili peppers an earlier application of VIGS at the germinating stage cotyledons was more efficient, VIGS infiltration was applied at the cotyledon stage. Thus, the emerging cotyledons of a total of 30 plantlets of cv. "Round" were agro-infiltrated with pTRV1 and pTRV2-*CaOvate *sense or pTRV2-*CaOvate *antisense. As a control, two more mock plants of the same cultivar at the same developmental stage were agro-infiltrated with pTRV1 and pTRV2 (empty vector). Approximately 9 weeks after the infiltration and while no changes were observed in the control mock plants infiltrated with the empty vector, one plant infiltrated with pTRV2-*CaOvate *sense (from now on referred to as "infiltrated plant 1") began to show changes in all its fruits' shaping becoming more oblong than the wild type (WT) fruits (see below). A second plant infiltrated with pTRV2-*CaOvate *antisense (infiltrated plant 2) exhibited varying dispersal of silencing effects in its fruits on different branches i.e. more oblong fruits in one branch next to wild type fruits in another branch, confirmed again by phenotypic measurements (Additional File 6). Thus only infiltrated plant 1 with a catholic elongation in all its fruits was chosen to be further characterized in more detail.

**Figure 4:**
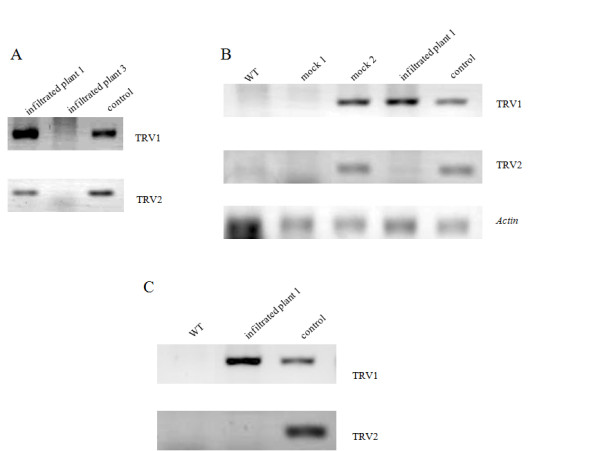
**RT-PCR detection of TRV1 and TRV2 viral RNAs**. A) In small fruits of approximately 10 DAA of infiltrated plant 1, with the changed shape phenotype and infiltrated plant 3, with the typical round shape phenotype, 9 weeks after infiltration. White - photobleached leaves from pepper plants infiltrated with pTRV2-Nb*Pds *were used as the control for the verification of the PCR success. TRV's transcription is confirmed by the presence of TRV1 and TRV2 transcripts in the infiltrated plant 1, while no TRV is detected in infiltrated plant 3. B) In leaves of the wild type (WT) - not infiltrated plant, of mock plant 1 and mock plant 2, of infiltrated plant 1, with the changed shape pheonotype approx. 11 weeks after infiltration. TRV1 transcripts are detected in mock 2 and infiltrated plant 1 but not in mock 1 and the WT. On the other hand, TRV2 transcripts are detected only in mock 2. Again white - photobleached leaves from pepper plants infiltrated with pTRV2-Nb*Pds *were used as the control for the verification of the PCR success. Pepper *Actin *was used for the verification of successful first strand cDNA synthesis. C) In 5 DAA fruits of the WT - not infiltrated plant and of infiltrated plant 1, 16.5 weeks after infiltration. TRV1 but not TRV2 transcripts are detected (as in leaves earlier) in the fruit of the infiltrated plant 1 while no transcripts are detected in the fruit taken from the WT.

To verify that the transcripts of the genomic RNA of TRV1 and TRV2 were present and diffused inside the infiltrated plant 1, showing uniformly the effects on the whole upper plant part, total RNA was extracted from small fruit (approx. 10 DAA) of this plant that although in the early stages of development, it was exhibiting an obvious change in its shape. Total RNA was extracted, also, from small fruit at the same developmental stage of another plant, from now on referred to as "infiltrated plant 3" that despite the fact that was infiltrated with pTRV2-*CaOvate *sense it did not show a change in the phenotype of its fruits. As shown in Figure 4A, transcripts of TRV1 and TRV2 were detected, through RT-PCR, in the small fruit of the infiltrated plant 1 but not in the small fruit of the infiltrated plant 3, while no amplification products were detected in the "NO reverse transcriptase" and "NO template" negative controls.

Later on, at approximately 11 weeks after infiltration, three to four whole leaves were also collected from the infiltrated plant 1, the plants infiltrated with the pTRV2 empty vector (mock controls) and the wild type (WT) - not infiltrated plant. The leaves from each of the plants separately were pooled together for total RNA extraction. As shown in Figure 4B, RT-PCR analysis confirmed the presence of TRV1 but not TRV2 transcripts in the leaves of the infiltrated plant 1. In the two mock plants tested, TRV1 and TRV2 transcripts were detected only in one of them (mock 2), while in the other neither transcript was detected (mock 1). Neither of the transcripts was detected in the wild type - not infiltrated plant. All the negative controls included resulted in no amplification products. Finally, 16.5 weeks after infiltration and while the infiltrated plant 1 kept on producing fruits with more oblong shape, 5 DAA fruit from the infiltrated plant 1 and from the not infiltrated, wild type control plant, were used for new total RNA extraction. Similar to results obtained with leaves analyzed 5 weeks earlier, transcripts of TRV1 but not TRV2 transcripts were detected, though RT-PCR analysis, in the 5 DAA fruit of the infiltrated plant 1 and neither of the viral transcripts was detected in the 5 DAA fruit of the not infiltrated, wild type plant, as it is shown in Figure 4C. All the negative controls included were free of amplification products.

**Figure 5:**
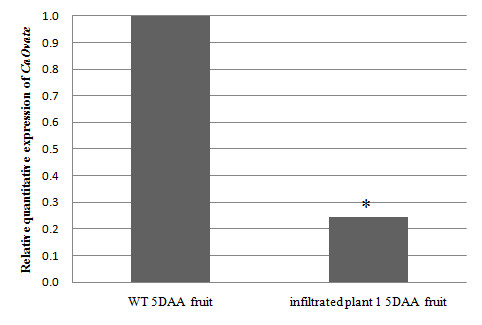
**Relative quantitative RT-PCR of *CaOvate *expression in fruit of 5 DAA from the wild type (WT) and the infiltrated plant 1**. First strand cDNA synthesis was accomplished starting from total RNA isolated from both fruits and using random hexamers and reverse transcriptase. This first strand cDNA was used in the PCR using gene-specific primers for *CaOvate*, one of them designed on the sequence outside the area covered by the construct. The samples from both plants were collected approx. 16.5 weeks after the infiltration which was done when the seedlings were in the stage of cotyledons. Asterisk indicates statistically significant difference (p < .05) of the expression levels of *CaOvate *in the 5 DAA fruit of the infiltrated plant 1 when compared to expression levels in the 5 DAA fruit of the WT. Pepper *Actin *was used as a reference gene.

**Figure 6:**
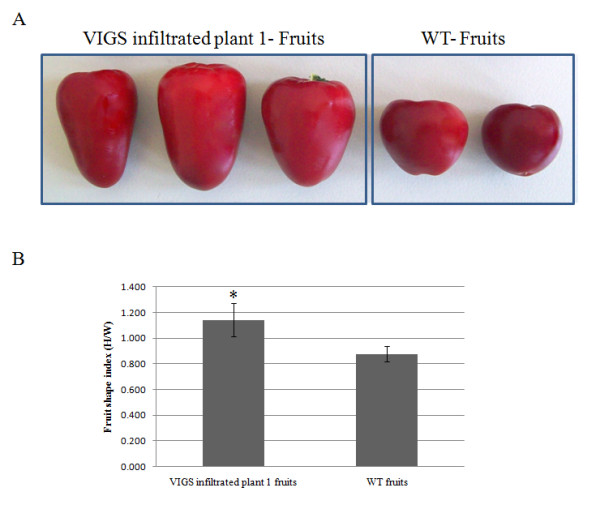
**Mature fruits collected from the infiltrated plant 1 and the WT plant and their phenotypic measurements**. A) Some characteristic mature fruits collected from the infiltrated plant 1 (left) and from the WT plant (right). B) Average fruit shape index of mature fruits of the wild type (WT) and of the VIGS infiltrated plant 1. The fruit shape index was calculated according to [1], as the ratio of highest fruit height to widest width. The fruits of the infiltrated plant 1 exhibit an average fruit shape index more than 1, characteristic of their oblong shape, while the average fruit shape index of the fruits of the WT is lower than 1. The difference between the two fruit shape indices is statistically significant (p < .05). Standard deviation bars are also shown.

Furthermore, more accurate relative quantitative RT-PCRs were performed for the relative quantitative determination of endogenous *CaOvate *mRNA levels in the 5 DAA fruit of infiltrated plant 1 and the wild type control. The primers used (OVATE FOR 4 and OVATE FINAL) were selected in such a way as to amplify a 415-bp fragment, part of which is not included in the VIGS construct (see *Methods*). This assay was allowing us to distinguish between the endogenous *CaOvate *mRNAs and the viral derived ones. The results showed a statistically significant (p < .05) decrease of about 75% in the levels of *CaOvate *expression in the 5 DAA fruit adopting a different, more oblong shape, in comparison to *CaOvate *expression in the 5 DAA fruit of round shape taken from the wild type (Figure 5). This reduction in the *CaOvate *levels in 5DAA fruit of the infiltrated plant 1 in comparison to the wild type control supports the conclusion that the observed changed phenotype in infiltrated plant 1 fruits compared to the phenotype of the WT's fruits (Figure 6A) is attributed to the successful silencing of *CaOvate *gene by VIGS. The phenotypic measurements in the mature fruits of the infiltrated plant 1 showed a significant change in fruits' length compared to that of the wild type. Specifically, the average fruit shape index is 1.14 for the fruits of the infiltrated plant 1 while the average fruit shape index of the fruits of the WT is 0.88 (Figure 6B). This statistically significant (p < .05) increase in the fruit shape index confirms the observation done macroscopically that the fruits of the successfully silenced plant are longer than the WT's.

### Expression of *CaGA20ox1 *in VIGS plant

**Figure 7:**
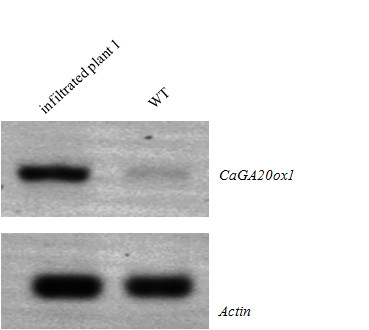
**RT-PCR analysis of *CaGA20ox1 *expression in fruit of 5 DAA from the infiltrated plant 1 and the wild type (WT)**. The samples from both plants were collected approximately 16.5 weeks after the infiltration which was done when the seedlings were in the stage of cotyledons. Pepper *Actin *was used as a reference gene. An obvious change is illustrated between the *CaGA20ox1 *expression in the two fruits: there is an increase in the expression of the gene in the 5 DAA fruit of the infiltrated plant compared to the expression in the 5 DAA fruit of the WT.

Since tomato's *Ovate *and *AtOFP1 *hold back growth [2,5] as a result of abridged cell elongation, due to their effect on gibberellin biosynthesis [5], we cloned and characterized *CaGa20ox1 *(data not shown) and studied its expression, by means of RT-PCR, in WT pepper plants of cv. "Round", as well as in the infiltrated plant 1 with the reduced *CaOvate *expression. As shown in Figure 7, our results suggest that there is an increase in the expression of *CaGA20ox1 *in the 5 DAA fruit of the infiltrated plant 1 comparing to the 5 DAA fruit of the WT, implying that *CaGA20ox1 *expression is affected by *CaOvate *in pepper.

## Discussion

In tomato, it was shown that the elongated fruit shape is specified mainly by four loci: *Ovate, Sun, Tri2.1/Dblk2.1 *and *Fs8.1*, with the first two segregating in some cultivars. However, it is the interaction between all the aforementioned loci that may control the fate of tomato fruit shape [1]. *Ovate *in particular is one of the two major loci (*Sun *is the other) responsible for the modulation of fruit shape, possibly determining the polarity of cell division early in floral development [3]. Comparative mapping analysis has shown that the tomato *Ovate *has orthologs in other Solanaceae species including pepper [42]. In particular, [43] suggest that there exists a small number of conserved QTLs that control fruit shape and size between tomato and pepper. They first identified a pepper fruit-shape QTL, *Fs 2.1*, localized in the tomato *Ovate *gene but having a comparatively lesser effect. More significantly however, they also identified a major fruit-weight QTL in pepper, *Fw 2.1*, found to be encoded by or tightly linked to *Ovate *[43], suggesting that *Ovate *may control fruit characteristics in pepper differently to tomato. The tight co-localization of tomato *Ovate *gene with pepper QTLs for a number of loci related to fruit diameter and shape, suggests a strong synteny and close relationship between the genes that control cell division, elongation and polarity [44]. For understanding fruit shape formation, we start in this work from *CaOvate *and one of its targets, a *GA20ox1 *gene designated as *CaGA20ox1*.

The analysis of the *CaOvate *genomic sequences obtained from the two cultivars studied showed that sequences differ in a SNP in the first exon of the gene, leading to a Threonine_Long _- to - Serine_Round _polymorphism in the resulting predicted amino-acid sequence. A C terminal DUF623 domain was identified on the predicted amino-acid CaOVATE sequence, a domain which exists in all AtOFPs and Solaneceaous OVATE proteins as well as in other uncharacterized proteins in other plants. This domain in *Arabidopsis*, as shown for AtOFP1 and AtOFP5, was found to help the contact with the BELL and KNOX homeodomains, regulating their subcellular localization [10] while in tomato it is the abolishment of this domain that causes the differences in fruit shape [2]. The bioinformatics analysis of all DUF623 domain sequences from Pfam enabled their segregation into subfamilies (Figure 2). The DUF623 domain of the CaOVATE was categorized in the same subfamily as other Solanaceous plants and the DUF623 domains of AtOFP7, AtOFP8 and AtOFP6. AtOFP7 was found to exhibit analogous function to AtOFP1, which is a known transcriptional repressor of *AtGA20ox1 *[5]. AtOFP1 is categorized in another subfamily along with other well characterized proteins such as AtOFP2, AtOFP3, AtOFP5 and an OVATE-like protein from rice. AtOFP5 was shown to be important for normal development and cell pattern in the *Arabidopsis *embryo sac [15]. The two subfamilies, the one with CaOVATE, AtOFP6, AtOFP7 and AtOFP8 and the other with AtOFP1, AtOFP2, AtOFP3 and AtOFP5, have a significant number of common amino-acids inside the domain. According to the specificity determining residues analysis, the two subfamilies have consistently differing amino-acids in positions 23 and 49 of the alignment (Figure 2) but the possible similar functions between the OFPs such as AtOFP1 and AtOFP7 [5] categorized in the two subfamilies may suggest that these changes do not alter the function of the domain, although they concern amino-acids that are not biochemically similar. In other words, it is possible that subfamilies 6 and 8 contain proteins acting similarly in plant growth and development, therefore placing our CaOVATE in a group of proteins that have been shown to participate in cell size and fruit shape determination in many plant species.

In tomato, what determines the shift from a round to a pear-shaped cultivar is a stop codon in the second exon of the *Ovate *sequence that puts an end in the translation of the mRNA to protein in the pear-shaped cultivar [2]. We were therefore unable to identify a similar mechanism in our two pepper cultivars.

We then examine whether different quantitative expression levels exist between the two pepper cultivars. The expression analysis of *CaOvate *showed there is a timing difference in the expression of the gene between the two pepper cultivars of different fruit shape, with cv. "Round" exhibiting a delay accompanied by increased expression compared to cv. "Long" (Figure 3). More specifically, in cv. "Round", the peak of *CaOvate *expression is observed after anthesis, in 5 DAA developing fruits. This is similar to tomato TA496, a round-fruited cultivar, in which the highest expression of *Ovate *was detected also after anthesis in a developing fruit of 4 DAA [2]. In cv. "Long" however, the peak of *CaOvate *expression is observed before anthesis as in tomato's cv. Yellow Pear (TA503), the final pear-shaped fruit of which is already evident in ovaries before anthesis when *Ovate *expression reaches its highest level. After anthesis of cv. Yellow Pear, *Ovate *expression drops sharply as also observed in pepper cv. "Long" [2]. These results may suggest that our two pepper cultivars exhibit quantity and timing differences in *CaOvate *expression which affect fruit shape. Finally, in tomato, the difference in the transcript levels of *Ovate *between the two cultivars with the different fruit shape is likely attributed to a 16-bp indel in the 5' upstream region [2]; in the pepper cultivars examined here no such difference was observed in the sequences of the 5' upstream region.

Silencing of *CaOvate *through VIGS in small plantlets of cv. "Round", resulted in plant that was further analyzed due to the altered phenotype exhibited in all of its fruits. More specifically, the fruits that this plant produced were more oblong and varied compared to the cultivar's typical round shape according to phenotypic measurements (Figure 6B). At 16.5 weeks after infiltration, by which time there were several fruits with changed phenotype on this particular plant, 5 DAA developing fruit was analyzed initially for the presence of TRV transcripts. It was shown that at the time of sampling, TRV1 but not TRV2 transcripts were detected, although both transcripts were detected a few weeks earlier in the same plant. This difference in detecting the viral transcripts throughout the organs and tissues of the infiltrated plant is likely attributed to the fact that only small RNAs produced by the viral transcripts travel inside the infiltrated plant. In the mock treated plants, i.e. plants treated only with TRV1 and TRV2 empty vectors, neither viral transcripts were detected, a finding in agreement with previous ones by [45] attributed to the relatively minimal infection speed of the TRV1 and TRV2 constructs in fully developed plants. Next, the expression level of the endogenous *CaOvate *gene was assessed through RT-PCR, and a statistically significant down-regulation of about 75% compared to the wild type 5 DAA fruit was detected. It is possible then that this down-regulation of *CaOvate *is involved in the alteration of the shape of fruits in cv. "Round" adopting a more elongated shape, consistent with previous findings describing *Ovate *as a growth suppressor [2]. In other words, the down-regulation of *CaOvate *accompanied by an increase in expression of its possible target, *CaGA20ox1*, are potentially the promoters of growth and thus fruit elongation in pepper. We should note that the silencing of the DUF623 domain through the application of the VIGS technique might have led to the silencing of other genes encoding for proteins that contain the domain in pepper (if any).

## Conclusions

Our work involved the cloning and characterization of a pepper gene, *CaOvate*, likely involved in the control of an important trait character, fruit shape, known to be affected by the widely applied technique of grafting. The *CaOvate *gene was cloned and sequenced from two pepper cultivars with different fruit shape (cv. "Round" and cv. "Long"). *CaOvate *encodes a protein that includes the DUF623 domain, characteristic of OFPs. Bioinformatics analysis placed CaOVATE in the same protein subfamily with functionally equivalent proteins from Solanaceae plants, including tomato, and three OFP members from *Arabidopsis*: AtOFP6, AtOFP7 and AtOFP8. We also studied the transcript levels of the gene during the development of flower and fruit in the two cultivars and statistically significant differences were observed, differences not only in quantity but also in timing; the expression of *CaOvate *was highest after anthesis in cv. "Round" and before anthesis in cv. "Long". Additionally, the successful down-regulation of *CaOvate *by VIGS in cv. "Round" plants, resulted in an obviously more oblong fruit shape in infiltrated plants. The transcript levels of the *CaGA20ox1 *- a target-gene of *CaOvate - *were also affected.

Overall, we have provided significant *in vivo, in vitro *and *in silico *evidence that we have successfully isolated *Ovate*-like genes from two pepper cultivars. *CaOvate *is likely involved in the determination of fruit shape. Work is in progress to study additional pepper genes homologues to respective tomato genes involved in controlling tomato fruit shape. We believe that our work will help us to understand better the molecular mechanisms involved in controlling pepper fruit shape as well as the evolutionary conservation of these mechanisms among Solanaceae and other more distant species. Furthermore, since in grafted vegetables the effect of rootstocks on scion fruit quality is well documented [46,47] including effects in pepper fruit shape [19-21], the work described herein could facilitate the understanding of mechanism for graft induced changes in pepper fruits too.

## Authors' contributions

AT participated in the design of the study, carried out cloning, expression analyses, and sequence and phylogenetic analyses of the genes, the VIGS experiment, and prepared the manuscript. KP participated in cloning and expression analyses, in setting up the VIGS experiment and in the analysis of results. ND contributed in the bioinformatics analyses and assisted in drafting the manuscript. AST conceived the study, directed the project and participated in the analysis of the results and finally wrote the manuscript. All authors have read and approved the final manuscript.

## Supplementary Material

Additional file 1 **Supplementary table 1**. PDF table 1 - Primer sequences used in the experiments. Click here for file

Additional file 2 **Supplementary figure 1**. PDF figure 1 - Alignment of the *CaOvate *genomic sequences from cv. "Long" and cv. "Round" along with the genomic sequence of the BAC clone 215H17, identified due to its high similarity to tomato *Ovate*. The SNPs between the sequences are localized in position 419, which is inside the first exon of the gene while the other two, in positions 654 and 746, are located inside the one and only intron of the gene. All SNP positions are boxed. The alignment was generated using the ClustalW program and edited with Bioedit. Click here for file

Additional file 3 **Supplementary figure 2**. Word figure 2 - Phylogenetic analysis of the DUF623 domain from the OFPs from *Arabidopsis *and related protein sequences from diverse species, including species of the Solanaceae family. The tree was generated using the MAFFT algorithm. The position of the DUF623 domain from CaOVATE is highlighted. Click here for file

Additional file 4 **Supplementary table 2**. Word table 2 - A summary of the BLAST results retrieved from several EMBL plant nucleotide sequence databases (see Methods), with the *CaOvate *cDNA sequence as query. The results are presented by database (rows) and species (columns). New/Additional species that produced significant hits and were added in the analysis are highlighted in grey. Click here for file

Additional file 5 **Supplementary figure 3**. PDF figure 3 - A) Fruits of a VIGS infiltrated plant that was infiltrated in the stage of 4-5 true leaves (preliminary experiment). Despite the different developmental stage of the two fruits depicted in the image, it is obvious that the fruit on the left of the picture is adopting a more oblong shape than the fruit on the right of the picture that is typically round, B) Average fruit shape index of mature fruits of the wild type (WT) and of the VIGS infiltrated plant that was infiltrated in the stage of 4-5 true leaves (preliminary experiment). The fruit shape index was calculated as the ratio of highest fruit height to widest width. The fruits of the VIGS infiltrated plant exhibit an average fruit shape index of 1, while the average fruit shape index of the fruits of the WT is lower than 1. The difference between the two fruit shape indices is statistically significant (p < .05). Standard deviation bars are also shown. Click here for file

Additional file 6 **Supplementary figure 4**. PDF figure 4 **- **A) Some characteristic mature fruits collected from the infiltrated plant with the pTRV2-*CaOvate *antisense construct (down) and from the WT plant (up), B) Average fruit shape index of mature fruits of the wild type (WT) and of the VIGS infiltrated - with the antisense construct- plant (infiltrated plant 2) that was infiltrated in the stage of the cotyledons. The fruits of the infiltrated plant exhibit an average fruit shape index more than 1, while the average fruit shape index of the fruits of the WT is lower than 1. The difference between the two fruit shape indices is statistically significant (p < .05). Standard deviation bars are also shown. Click here for file
