# Supplementary material for: Multiple evidence for the role of an Ovate-like gene in determining fruit shape in pepper
Source: BMC Plant Biol. 2011 Mar 14;11:46. doi: 10.1186/1471-2229-11-46 (PMC3069956; doi:10.1186/1471-2229-11-46)
Supplement: Additional file 1 — Supplementary table 1. PDF table 1 - Primer sequences used in the experiments. [file 1471-2229-11-46-S1.PDF]

Primer	Sequence
AUAP[1]	5’-GGCCACGCGTCGACTAGTAC-3’
OVATE FOR 1	5’-GGGTGATTGTTGACGCTTT-3’
OVATE FOR 2	5’-ATGGGAAAAAGTTTGAACTTCG-3’
OVATE FINAL	5’-CCAGCATCTATGTTAGGTCTT-3’
OVATE REV 1	5’-CTTCAGAGACGAAGGAGCTG-3’
OVATE FOR 3	5’-CCAGAAGCTTCGATTTCTCG-3’
OVATE FOR 4	5’-ATCGTCGGTTTCTACATCA-3’
OVATE REV 2	5’-CCGACGATCTTCTACCTTTT-3’
OVATE FOR 5	5’-TATCACAGAGAGAGTTTTCGG-3’
GA20ox1 FOR 2	5’-GTGCCGTAGCACAAGAACTTC-3’
GA20ox1 REV 2	5’-CTTGATAAATATTCCCAAGATGGC-3’
ACTFW (adapted from [36])	5’-ATCCCTCCACCTCTTCACTCTC-3’
ACTRV (adapted from [36])	5’-GCCTTAACCATTCCTGTTCCATTATC-3’
TRV1 FOR	5’-ATTGAGGCGAAGTACGATGG- 3’
OYL 198 REV	5’-GTAAAATCATTGATAACAACACAGACAAAC-3’
Coat Protein FOR	5’-CGGGCTAACAGTGCTCTTG-3’
Coat Protein REV	5’-CTCCCTTGGTTCGTCGTAAC-3’

[1] Abridged Universal Anchor Primer
