# Supplementary material for: Multiple evidence for the role of an Ovate-like gene in determining fruit shape in pepper
Source: BMC Plant Biol. 2011 Mar 14;11:46. doi: 10.1186/1471-2229-11-46 (PMC3069956; doi:10.1186/1471-2229-11-46)
Supplement: Additional file 2 — Supplementary figure 1. PDF figure 1 - Alignment of the CaOvate genomic sequences from cv. "Long" and cv. "Round" along with the genomic sequence of the BAC clone 215H17, identified due to its high similarity to tomato Ovate. The SNPs between the sequences are localized in position 419, which is inside the first exon of the gene while the other two, in positions 654 and 746, are located inside the one and only intron of the gene. All SNP positions are boxed. The alignment was generated using the ClustalW program and edited with Bioedit. [file 1471-2229-11-46-S2.PDF]

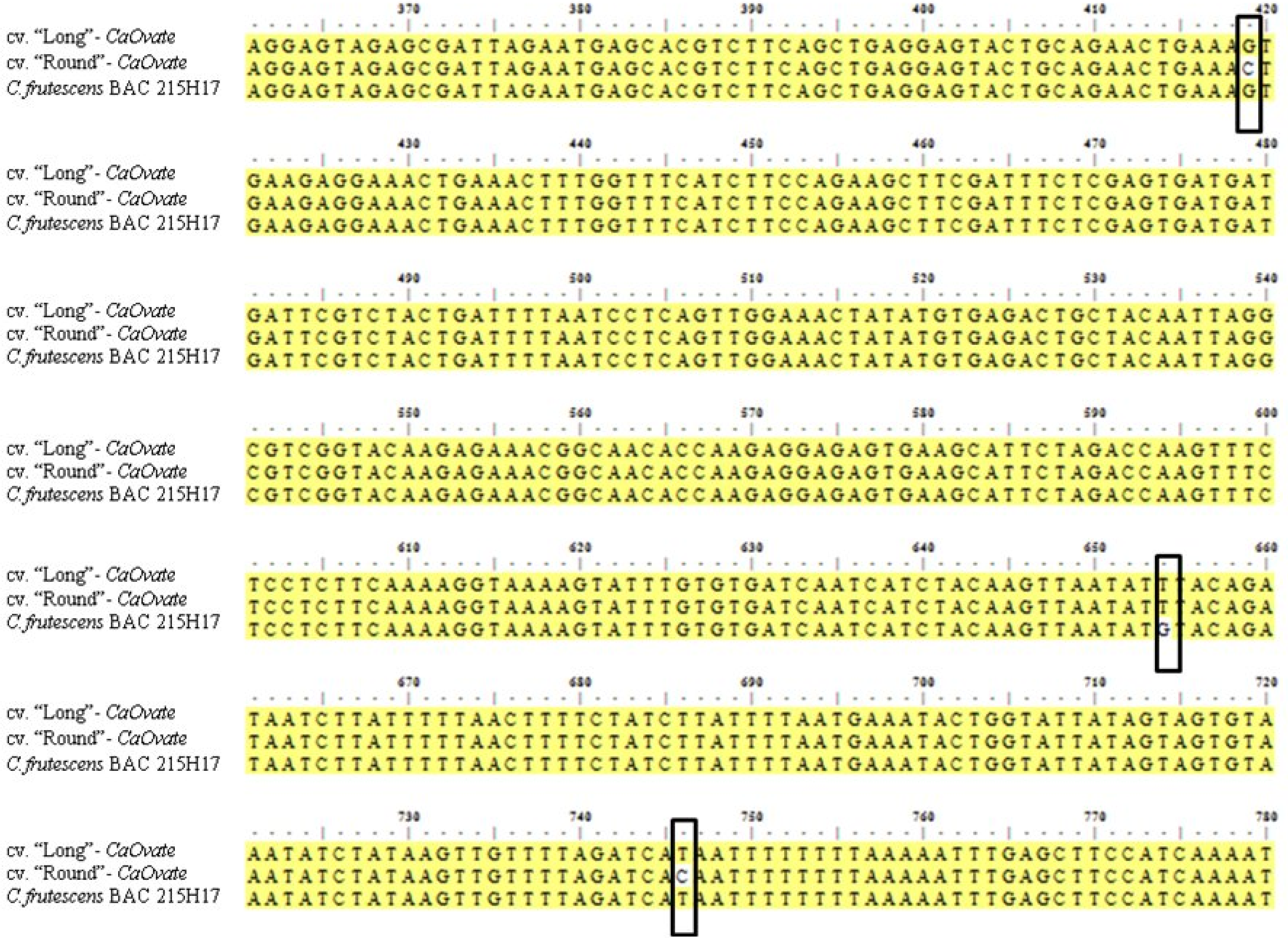

370 380 390 400 410 420
cv. "Long"- CaOvate AGGAGTAGAGCGATTAGAATGAGCACGTCTTCAGCTGAGGAGTACTGCAGAACTGAAAGT
cv. "Round"- CaOvate AGGAGTAGAGCGATTAGAATGAGCACGTCTTCAGCTGAGGAGTACTGCAGAACTGAAACT
C. frutescens BAC 215H17 AGGAGTAGAGCGATTAGAATGAGCACGTCTTCAGCTGAGGAGTACTGCAGAACTGAAAGT
430 440 450 460 470 480
cv. "Long"- CaOvate GAAGAGGAAACTGAAACTTTGGTTTCATCTTCCAGAAGCTTCGATTTCTCGAGTGATGAT
cv. "Round"- CaOvate GAAGAGGAAACTGAAACTTTGGTTTCATCTTCCAGAAGCTTCGATTTCTCGAGTGATGAT
C. frutescens BAC 215H17 GAAGAGGAAACTGAAACTTTGGTTTCATCTTCCAGAAGCTTCGATTTCTCGAGTGATGAT
490 500 510 520 530 540
cv. "Long"- CaOvate GATTCGTCTACTGATTTTAATCCTCAGTTGGAAACTATATGTGAGACTGCTACAATTAGG
cv. "Round"- CaOvate GATTCGTCTACTGATTTTAATCCTCAGTTGGAAACTATATGTGAGACTGCTACAATTAGG
C. frutescens BAC 215H17 GATTCGTCTACTGATTTTAATCCTCAGTTGGAAACTATATGTGAGACTGCTACAATTAGG
550 560 570 580 590 600
cv. "Long"- CaOvate CGTCGGTACAAGAGAAACGGCAACACCAAGAGGAGAGTGAAGCATTCTAGACCAAGTTTC
cv. "Round"- CaOvate CGTCGGTACAAGAGAAACGGCAACACCAAGAGGAGAGTGAAGCATTCTAGACCAAGTTTC
C. frutescens BAC 215H17 CGTCGGTACAAGAGAAACGGCAACACCAAGAGGAGAGTGAAGCATTCTAGACCAAGTTTC
610 620 630 640 650 660
cv. "Long"- CaOvate TCCTCTTCAAAAGGTAAAAGTATTTGTGTGATCAATCATCTACAAGTTAATATTTACAGA
cv. "Round"- CaOvate TCCTCTTCAAAAGGTAAAAGTATTTGTGTGATCAATCATCTACAAGTTAATATTTACAGA
C. frutescens BAC 215H17 TCCTCTTCAAAAGGTAAAAGTATTTGTGTGATCAATCATCTACAAGTTAATATGTACAGA
670 680 690 700 710 720
cv. "Long"- CaOvate TAATCTTATTTTTAACTTTTCTATCTTATTTTAATGAAATACTGGTATTATAGTAGTGTA
cv. "Round"- CaOvate TAATCTTATTTTTAACTTTTCTATCTTATTTTAATGAAATACTGGTATTATAGTAGTGTA
C. frutescens BAC 215H17 TAATCTTATTTTTAACTTTTCTATCTTATTTTAATGAAATACTGGTATTATAGTAGTGTA
730 740 750 760 770 780
cv. "Long"- CaOvate AATATCTATAAGTTGTTTTAGATCATAATTTTTTTTAAAAATTTGAGCTTCCATCAAAAT
cv. "Round"- CaOvate AATATCTATAAGTTGTTTTAGATCACAATTTTTTTTAAAAATTTGAGCTTCCATCAAAAT
C. frutescens BAC 215H17 AATATCTATAAGTTGTTTTAGATCATAATTTTTTTTAAAAATTTGAGCTTCCATCAAAAT
