# Supplementary figures and images for: Multiple evidence for the role of an Ovate-like gene in determining fruit shape in pepper
Source: BMC Plant Biol. 2011 Mar 14;11:46. doi: 10.1186/1471-2229-11-46 (PMC3069956; doi:10.1186/1471-2229-11-46)

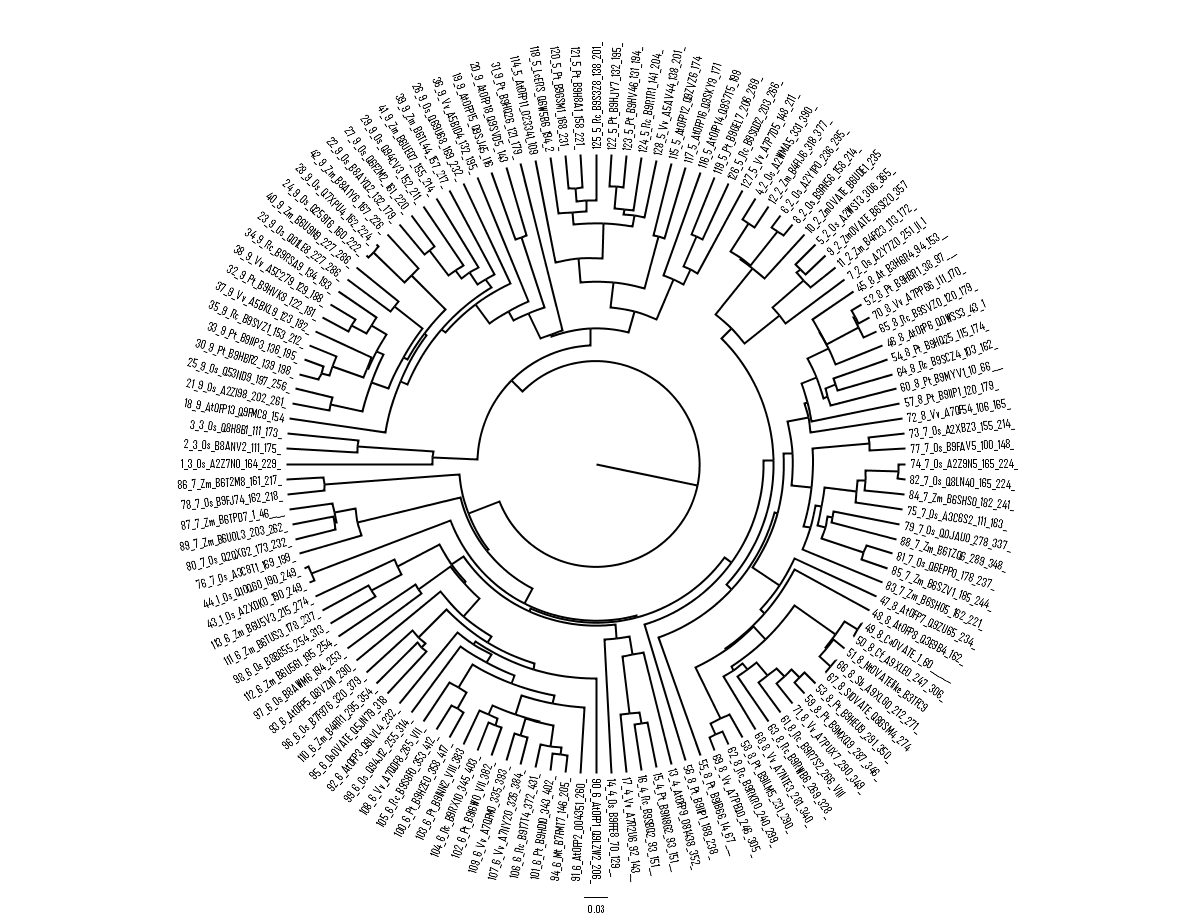

Supplement: Additional file 3 — Supplementary figure 2. Word figure 2 - Phylogenetic analysis of the DUF623 domain from the OFPs from Arabidopsis and related protein sequences from diverse species, including species of the Solanaceae family. The tree was generated using the MAFFT algorithm. The position of the DUF623 domain from CaOVATE is highlighted. [file 1471-2229-11-46-S3.DOC]

A

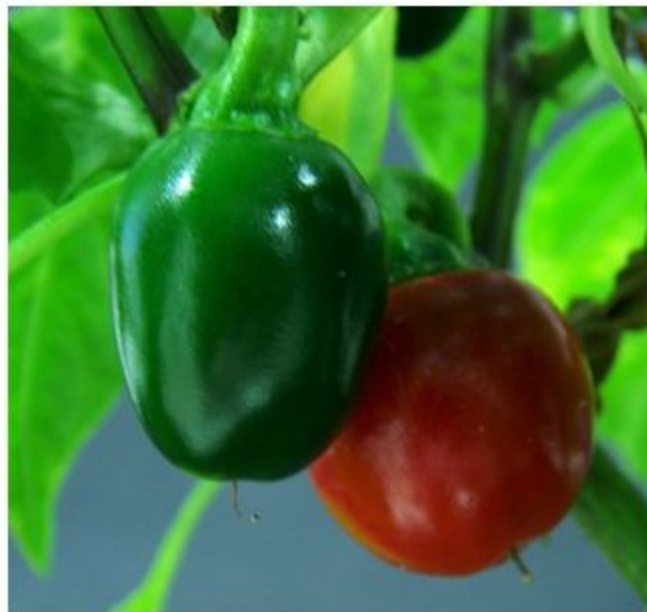

B

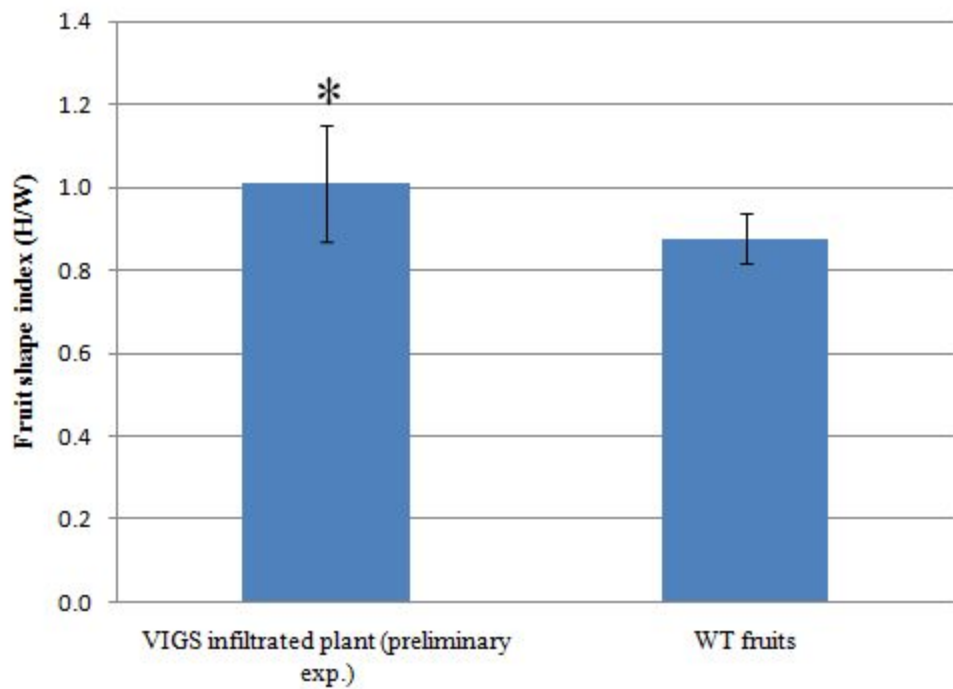

Supplement: Additional file 5 — Supplementary figure 3. PDF figure 3 - A) Fruits of a VIGS infiltrated plant that was infiltrated in the stage of 4-5 true leaves (preliminary experiment). Despite the different developmental stage of the two fruits depicted in the image, it is obvious that the fruit on the left of the picture is adopting a more oblong shape than the fruit on the right of the picture that is typically round, B) Average fruit shape index of mature fruits of the wild type (WT) and of the VIGS infiltrated plant that was infiltrated in the stage of 4-5 true leaves (preliminary experiment). The fruit shape index was calculated as the ratio of highest fruit height to widest width. The fruits of the VIGS infiltrated plant exhibit an average fruit shape index of 1, while the average fruit shape index of the fruits of the WT is lower than 1. The difference between the two fruit shape indices is statistically significant (p < .05). Standard deviation bars are also shown. [file 1471-2229-11-46-S5.PDF]

A

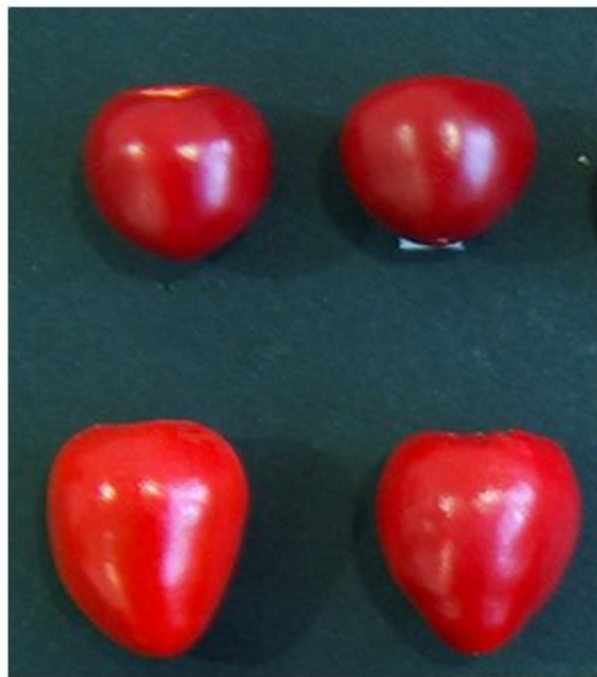

B

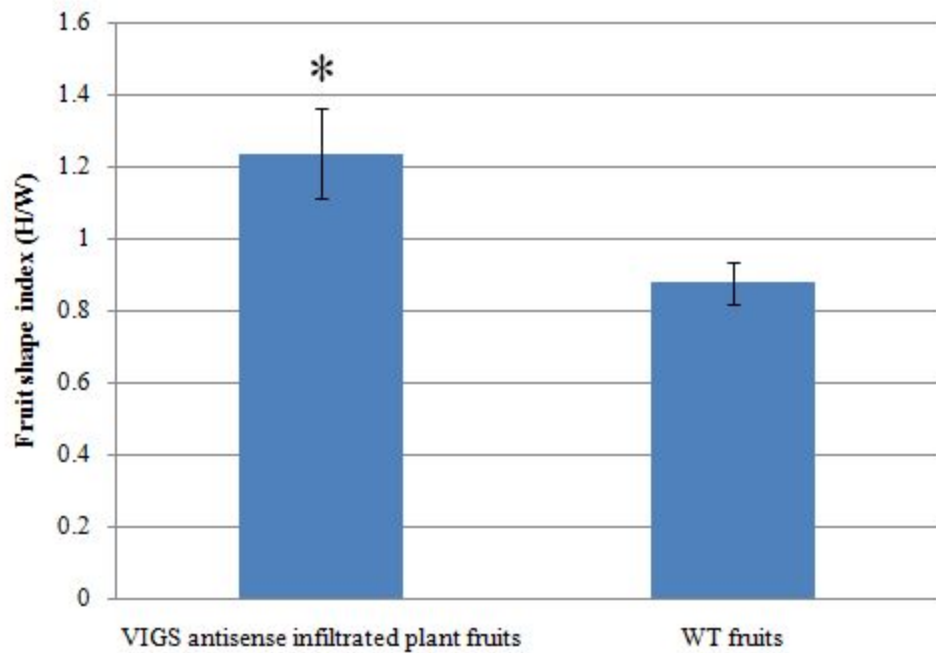

Supplement: Additional file 6 — Supplementary figure 4. PDF figure 4 - A) Some characteristic mature fruits collected from the infiltrated plant with the pTRV2-CaOvate antisense construct (down) and from the WT plant (up), B) Average fruit shape index of mature fruits of the wild type (WT) and of the VIGS infiltrated - with the antisense construct- plant (infiltrated plant 2) that was infiltrated in the stage of the cotyledons. The fruits of the infiltrated plant exhibit an average fruit shape index more than 1, while the average fruit shape index of the fruits of the WT is lower than 1. The difference between the two fruit shape indices is statistically significant (p < .05). Standard deviation bars are also shown. [file 1471-2229-11-46-S6.PDF]
