# Supplementary material for: Multiple evidence for the role of an Ovate-like gene in determining fruit shape in pepper
Source: BMC Plant Biol. 2011 Mar 14;11:46. doi: 10.1186/1471-2229-11-46 (PMC3069956; doi:10.1186/1471-2229-11-46)
Supplement: Additional file 4 — Supplementary table 2. Word table 2 - A summary of the BLAST results retrieved from several EMBL plant nucleotide sequence databases (see Methods), with the CaOvate cDNA sequence as query. The results are presented by database (rows) and species (columns). New/Additional species that produced significant hits and were added in the analysis are highlighted in grey. [file 1471-2229-11-46-S4.DOC]

|  | ***N. tabaccum* (tobacco)** | ***S. lycopersicum* (tomato)** | ***S. bulbocastanum* (wild potato)** | ***S.melongena* (eggplant)** | ***S.phureja*** | ***S.chacoense* (chaco potato)** | ***C.frutescens* (Tabasco pepper)** |
| --- | --- | --- | --- | --- | --- | --- | --- |
| **EST** | EM_EST:AM844325 EM_EST:AM819284 EM_EST:AM830327 EM_EST:FG196672 EM_EST:BP528963 | EM_EST:BM412343 |  | EM_EST:FS017096 EM_EST:FS014673 EM_EST:FS085426 |  | EM_EST:DN981583 |  |
| **GSS** | EM_GSS:ET989388 EM_GSS:FH073325 EM_GSS:FH520404 EM_GSS:ET750080 EM_GSS:FH602496 EM_GSS:FH145906 EM_GSS:FH707981 EM_GSS:FI059322 EM_GSS:FH602427 EM_GSS:FH012050 EM_GSS:FH012060 EM_GSS:FI068807 |  |  |  | EM_GSS:GS670300 |  |  |
| **HTC** |  | EM_HTG:AK247861 |  |  |  |  |  |
| **CDS** | EMBLCDS:ABW05088 | EMBLCDS:AAN17752  EMBLCDS:AAG01121 | EMBLCDS:ABU45215 |  |  |  | EMBLCDS:ABU45184 |
| **STD** | EM_PL:EU043369 | EM_PL:AY140893 EM_PL:AF273333 EM_PL:AC226504 EM_PL:AC215492 | EM_PL:EF517794 |  |  |  | EM_PL:EF517792 |

Additional File 4

**Table 2. A summary of the BLAST results retrieved from several EMBL plant nucleotide sequence databases with the *CaOvate* cDNA sequence as query. The results are presented by database (rows) and species (columns). New, compared to the primary bioinformatics analysis, species that produced significant hits and were added in the analysis are highlighted in grey. EST: expressed sequence tags, GSS: genome survey sequences, HTC: high throughput cDNA sequencing, HTG: high throughput genome sequencing, CDS: Coding sequences and STD: Standard - all entries not classified as above).**
